# The blacksmith approach: a strategy for teaching and learning in the medical anatomy course (a qualitative study)

**DOI:** 10.1186/s12909-022-03800-1

**Published:** 2022-10-20

**Authors:** Arash Shojaei, Amin Feili, Javad Kojuri, Ali Norafshan, Leila Bazrafkan

**Affiliations:** 1 School of Medicine, Shiraz University of Medical Sciences, Shiraz, Iran; 2 Clinical Education Research Center, Shiraz University of Medical Sciences, Shiraz, Iran, Shiraz University of Medical Sciences, Shiraz, Iran; 3 Histomorphometry and Stereology Research Center, Department of Anatomy, School of Medicine, Shiraz University of Medical Sciences, Shiraz, Iran; 4 Clinical Education Research Center, Shiraz University of Medical Sciences, Shiraz, Iran, Shiraz University of Medical Sciences, Shiraz, Iran

**Keywords:** Anatomy education, Teaching, Learning, Medical faculty, Medical students, Qualitative

## Abstract

**Background:**

Anatomy is a symbolic, essential core topic and one of the fundamental pillars of medical and paramedical knowledge. Nevertheless, few exploratory data analyses have focused on how students approach learning anatomy. This study examined how students perceive their learning experience during anatomy lessons and how to make a model which promotes their meaningful learning and professional identity.

**Methods:**

Using purposive sampling with maximum variation, we conducted a qualitative content analysis at the Shiraz University of Medical Sciences in Iran (2020 to 2021). Twenty-four medical students and twelve faculty members of Iran’s medical science universities were enrolled in the study. The data were collected through semi-structured interviews and analyzed according to the theme.

**Results:**

A conceptual model emerged from the data analysis with the main theme called ***the blacksmith approach***, which included Three sub-themes: (1) making a new forge (adequate preparation and mindful beginning), (2) heating the students’ hearts (considering supporting systems that learners need) and (3) using Sledgehammer’s approach (teaching anatomy by using more active methods and engaging all neuroanatomical regions) and (Using fun for enjoyable learning). All the concepts were related to each other.

**Conclusion:**

Medical students experience a challenging fundamental evolution into professional doctors. Educational systems focus primarily on teaching and learning, while students’ transition can be facilitated by a three-step model called the Blacksmith Approach. It best serves as an educational framework for any pivotal, preclinical course capable of helping students acquire new roles and tackle challenges. Further research should be conducted to confirm how hard work leads to satisfying results with the opportunity to create enjoyable learning.

**Supplementary Information:**

The online version contains supplementary material available at 10.1186/s12909-022-03800-1.

## Introduction

Anatomy, as a branch of biological and medical sciences, is one of the introductory courses in the learning experience of undergraduate medical students, which is essential for impending clinical practice and clinical proficiency [1, 2]. A challenging question facing medical education is how to change some young students into responsible and resilient professional physicians. Most of the answers are being placed in clinical years; however, we can begin the transition process earlier with a promising tool called anatomy [3, 4].

Despite being one of the fundamental pillars of medical knowledge, anatomy has been pulled back as a simple course in the preclinical period. Historically, ancient medical schools have relied heavily on philosophical and intuitive systems of thought [5]. Experimental investigations by dissection helped physicians actualize the human body as a combination of multiple organ systems. [6]. We can assume that anatomy science has led to reform in how medicine views the human being from a spiritual, holistic being into a biopsychosocial person. However, anatomy has been underestimated in current medical pedagogy [7, 8].

Recent evidence indicates that we can reform anatomy courses making them as influential as they can be. [9–11]. Anatomy and dissection sessions can act as students’ first encounters with the humanized face of medicine, where medical students can learn teamwork, morality, coping strategies, and communication skills. In addition, anatomy can be perceived as a way of internalizing self-awareness, medical epistemology, empathy, and medical ethics [6, 12, 13].

While a myriad of teaching approaches are available for anatomy education, they have changed according to the needs of learners or global crises like covid-19. The primary approaches include lecture, dissection, and demonstration of cadavers. In addition, many schools incorporated radiological imaging as a learning tool for teaching anatomy in the living body. Considering the shortcomings of traditional methods, medical schools started implementing more innovative techniques that enhanced active learning. While Problem-based learning, flipped classroom, and reflective writing activities on the cadaveric dissection fostered communication skills and self-directed learning [12, 14, 15], other methods focused on students’ learning styles: drawing and whiteboarding, three-dimensional printing (3DP), and digital models for visual learners; play-doh, role modeling and peer physical exam for tactile learning; singing, dancing, yoga, and pilates for kinaesthetic learners [16–18]. The covid pandemic disrupted anatomy education as the number of donated cadavers decreased substantially, and social distancing prevented in-person classes or labs [17–19]. Accordingly, the teaching approaches relied, more than ever, on technology leading to the widespread use of computer-assisted learning, web-based learning via social media (esp. Facebook and youtube), and a more prominent role for Augmented-Reality and Virtual-Reality [20, 21]. The main focus behind all these methods and strategies is better teaching anatomy for its sake. However, such an approach does not allow us to use the full potential of anatomy science as a core element in changing young students into self-confident and resilient learners, which leads to the creation of future professional physicians [22, 23].

Physicians need professional competencies, a set of which essential competencies, including professional resilience, is and emotional competencies, to achieve goals. As a capacity to endure difficulties and quickly improve from a stress-inducing experience, resilience helps one to make valuable adaptations to problems and can utilize as a protective defense against adversities, thus facilitating welfare [23, 24]. Resilience is referred to as one’s capability to maintain and improve their well-being when faced with life challenges. Resilience can be considered a behavior acquired during training and various courses, including the main course of anatomy. Resilience includes cognitive processes and has four dimensions, including self-efficacy. Planning, Self-control, Commitment, and perseverance include the following: young doctors must be present in various educational environments and support clinical and educational supervisors in enduring difficulties to help them develop personal and professional resilience [24, 25].

In Iran and at Shiraz University, anatomy courses entail three steps. Initially, a topic is introduced through face-to-face, online or offline lectures with a slide or a short video clip. Next, students work on anatomical modeling and, finally, on a cadaver [26, 27].

Today, not only the medical students must bear a significant load of details in anatomy courses, which can reduce their motivation, but they should also face the educational consequences of the covid pandemic, which include loss of hands-on learning activities and limited access to tools like cadavers, models, pathology specimens, and skeletons [28–34]. Losing face-to-face contact and direct interaction with peers and teachers may hinder the students’ growth as future physicians [6, 13]. Therefore, finding ways to improve the learning experience is more challenging than ever.

## Objectives

In this study, we aimed to identify the themes that explain the strategy for teaching and learning and foster meaningful learning and professional identity in medical anatomy courses among medical students in Iran. Also, we aimed to develop a conceptual framework to explain this strategy development based on the experience of both students and faculty members(FM) through a qualitative study.

## Materials and methods

### Design

A qualitative approach was used by inductive content analysis in the first step [35]. Then we formulated the discovered themes into a comprehensive pedagogical approach.

### Setting

This study was conducted at Shiraz University of Medical Sciences (SUMS). The university currently includes more than 10,000 students, 200 majors, 782 faculty members, 54 research centers, 13 educational hospitals, a history of 70 years, and an assertive group teaching anatomy with fourteen faculty members in different academic ranks (assistant professors, associated professors, and full professors).

Medical students start an anatomy course as they enter medical school with an integrated organ-based curriculum. They pass 15 academic and practical units of this course, including complete systems: blood circulation, respiration, endocrine, musculoskeletal, and urinary, in integration with other classes such as physiology, biochemistry, and clinical courses on the same topics. Students participate in formative and summative assessment tests such as quizzes and final examinations to improve learning and obtain end-of-semester grades. Students should reveal in-depth, thoughtful principles of anatomy through multiple-choice questions(MCQ) tests. Also, Laboratory work examination includes dissecting preserved specimens, microscopic study, and computer simulations in objective structured clinical examination (OSCE) situations to reveal their competence in the anatomy courses [28, 36].

### Sampling

We used purposive and snowball sampling with maximum variation. Accordingly, we selected 24 medical students from year 1 to senior interns and 12 FMs within a wide range of academic rank and experience from 9 Iranian Universities of Medical Sciences in 2020–2021.

The inclusion criteria for medical students were studying in the first year of basic science to senior interns based on their willingness to participate. The inclusion criteria for faculty members in the anatomy or medical education field were a minimum of five-year teaching experience and a desire to participate. The exclusion criterion was the unwillingness to participate in the study. Initially, we collected data from a medical teacher well known for his high-quality teaching; then, we continued data gathering from medical students, anatomy faculty members, and medical education experts until data saturation was obtained and when no new code was obtained during interviews and repetition of the previous categories and codes [37–40]. Sampling and data coding continued till data saturation when no new code was obtained during interviews and repetition of the previous categories and codes.

### Data collection

We utilized semi-structured interviews. At first, we contacted participants by telephone to explain the purpose of the study and research questions. The interviews were done in a quiet place, at an appropriate time, face-to-face, and individually. The interview questions focused on participants’ experiences of learning and teaching anatomy. The interviews started with questions as follows:

- What is your description of successful teaching/learning in an anatomy course?
- Tell me about your experiences in learning/teaching anatomy and different situations, including the dissection laboratory.

Then, according to the participants’ answers, we asked exploratory questions. We searched for signs of success in teaching and learning, the factors that lead to failure, and the characteristics that can change the behavior and vision of medical students. Each interview took 25 to 80 min, with an average of 45 min.

Moreover, we gathered some field notes from the laboratory’s educational atmosphere. Data were collected and analyzed using Microsoft One Note2010, Microsoft, Redmond campus, US.

### Data analysis

We listened to each recorded interview to get an overall understanding. Then we analyzed verbatim and in-depth using inductive content analysis before the following interview, so each interview guided the next.

The meaning units consisted of words and sentences abstracted and labeled with codes. Twenty-six participants were interviewed in one session, and two or three sessions were conducted for six subjects.

The thematic content analysis includes six stages: [1] familiarizing with data, [2] generating the initial codes, [3] searching for themes, [4] reviewing the themes, (the various codes were compared based on similarities and differences in meaning and were categorized together) [5] defining and naming themes, and [6] preparing the report [41, 42].

After conducting iterative and comparative line-by-line coding procedures, we sorted similar codes into subcategories. Finally, we rearranged similar subcategories and domains into major categories [39].

In qualitative research, data quality and accuracy are used instead of validity and reliability. Lincoln and Guba have proposed four methods for validating data that many qualitative researchers have used: credibility, dependability, confirmability, and transferability, which will be discussed later [43, 44].

We used various methods to validate the study, such as prolonged involvement (15 months from July 2020 to Nov 2021) with the participants, data, and subjects. Additionally, we performed member checks and expert checks for coding. For the member check, we sent the results of each analysis to the very participant, who would confirm them based on the interview and their own experiences.

The analysis results and categories were shared, approved by the supervisors, and approved by an experienced qualitative researcher.

Transferability is defined by the generalizability of the results to fully describe the subject area and the characteristics of the participants with maximum variation in sampling. The generalization of the results was left to the reader to decide on the given information [45].

## Results

### Participants’ characteristics

In this study, semi-structured interviews were performed with 24 medical students from year 1 to senior interns, 15 (62.5% were males), 9 (37.5% were females), and 12 faculty members, 6 (50% were males), 6 (50% were females), with various academic ranks and experience from 9 (Shiraz, Tehran, Fasa, Jahrom, Bandarabas, Yasuj, Gerash, Larestan, and Bushehr) Iranian medical schools. The participants’ age ranged from 19 to 62 years old, with a mean of 33.4 ± 8.4 years. Based on the results, 12 students (50%) were in the clinical training period as students, externs, and interns, and 12 participants (50%) were in the basic sciences period. Accordingly, five participants (20%) had a history of being top students, and four (16.7%) had a history of dropout in anatomy courses. Furthermore, the work experience of the faculty members ranged from 5 to 35 years in teaching anatomy; 9(75%) anatomy faculty members and 3(25%) experts in medical education; faculty member participants, 3(25%) were single, and 9 (75%) were married. (Table 1)

**Table 1:** Demographic Characteristics of the Study Participants

Participants	Characteristics			
**students**	**Gender**	No	Percent (%)	**Mean Age (y ± SD)**
	Male	15	62.5	22.3 ± 8.4
	Female	9	37.5	
	**Degree**			
Faculty members	assistant professors,	3	25	37.4 ± 11.7
	associated professors	5	41.67	
	full professors	4	33.34	

From all the interviews, 334 codes were extracted. After the dismissal of similar codes and their integration, the experience of medical students was conceptualized into a central theme called ***the blacksmith approach***, which included Three sub-themes: (1) making a new forge (Adequate preparation and mindful beginning), (2) heating the students’ hearts (considering supporting systems that learners need) and (3) using a Sledgehammer’s approach (teaching Anatomy thorough engaging all neuroanatomical regions in the Age of Pandemics). All the concepts were related to each other and resulted in a pattern revealing the experience of students and faculty members on strategies for successful learning and teaching of anatomy lessons. The main themes, as well as subordinate themes, are listed in Table 2.

**Table 2:** The Blacksmith Approach: Best strategy for teaching and learning in the medical anatomy course. Themes, sub-themes, and codes

Main Themes	Sub Themes	code	cods
**Blacksmith Approach**	**Redesigning Educational Mindset (Making a New Forge)**	**Creating a Vision for Students**	√ Anatomy is hard, bulky, should be taken seriously √ I can help myself more than anyone √ integrated curriculum √ self-study / Self-confidence scarcely happens in the real world! √ professional identity √ prospective physicians
		**Mindful Clinical View**	
	**Role Modeling and Support (Heating up the Students’ Hearts)**	**Joyful Teaching (Telling Stories/Games/ Humor)**	√ every trick must be done √ enjoy class √ follow me in the classroom
		**Student Support System**	√ susceptible students √ assistance √ Vulnerable √ right supporters Sitting and talking to them
	**Sledgehammer Approach (Thorough Engagement in the Age of Pandemics)**	**Study Guide**	√ Preparedness √ Road map
		**Reference Autonomy**	√ self-learning √ self-direction
		**Neuroanatomical Engagement**	√ Visual Learning √ Auditory Learning √ Sensory Learning √ Brain Hemispheres Long-term Memorization
		**Mindful Dissection, Better Transition**	√ It smells so bad, but we need the observation √ We got used to it √ ethically important √ respect the dead body √ respect to the living body
		**Drawing**	√ easy to learn √ Draw a lot
		**Problem-Based Learning**	√ Look at the patient’s family √ Deep worry √ What is the next step of the problem?
		**Peer-Assisted Learning**	√ Peer to the peer group √ Discussing the problem

### Central theme: blacksmith approach

**Fig. 1:**
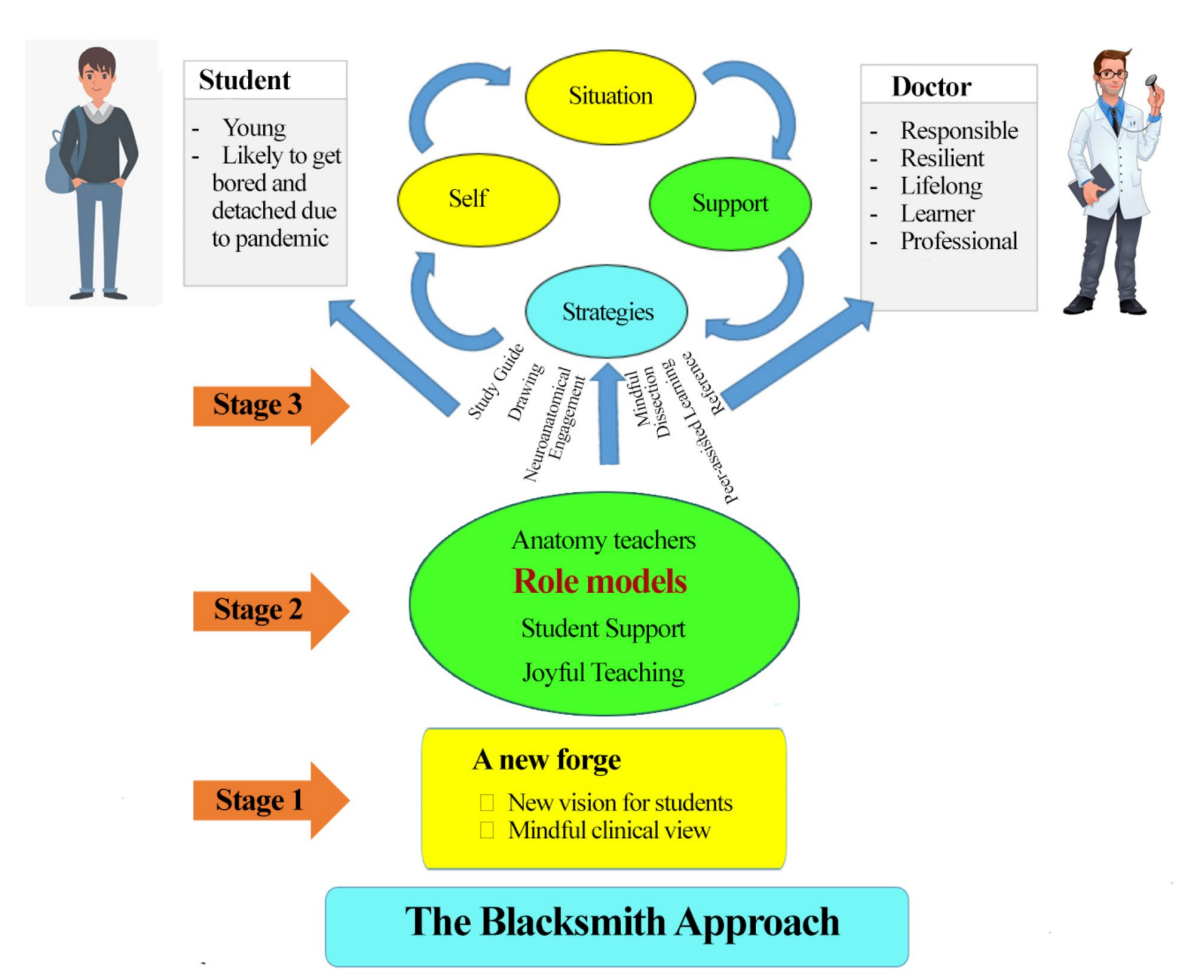
The role of the blacksmith approach in the stages of teaching and learning anatomy in medical students is based on the study’s results. First, the blacksmith (educational system) must prepare a new blacksmith (redesign the educational mentality and create a new perspective on anatomical knowledge in students) and prepare a new forge; then, he/she must heat the metal (supporting young students and having good role-modeling) and then shape the metal with a sledgehammer (the sledgehammer approach to using different teaching strategies and methods) to create a sword or self-confident and knowledgeable learner in medicine (professionalism)

According to the participants, in the student’s learning and maturity process, the teacher and the student act rationally and consciously and are aware of the factors that motivate and control them. Thus, they accept the risks, specific conditions, and hardships that must be tolerated. The student should handle this hardship to acquire a capacity to change, and the teacher should facilitate the change and their primary goal of teaching. In a sense, this is the same approach as the Blacksmiths. Blacksmithing symbolizes endurance, honor, and work under challenging conditions in our culture. (Fig. 1) We took the central theme from an interview with a professor who said:

”…*The Sledgehammer should be heavy. Its nature is heavy; otherwise, it will not be capable of smoothing any iron. The nature of some courses, such as anatomy, is challenging, but we must make it enjoyable for the student*….“ (FM no.6).

### Sub theme1: redesigning educational mindset (making a new forge)

Creating an educational context is the first step for young students to begin their transition in the learning process. This context was found to have two aspects as follows:

1. **Creating a Vision for Students**.

Some students mentioned a change in their mindset from the beginning with the idea that they have a difficult task ahead and should know what is helpful or not. In addition, realizing that the student is the most important person to help themselves seemed to provide a better sense of success.

According to the participants, anatomy is one of the most voluminous and complex courses in medicine, which students should take seriously from the beginning.

*“Anatomy is hard, bulky, and should be taken seriously.“ (Student* no. *9)*

Even though many have oversimplified it, successful students have attempted to learn it through vision and self-awareness.

*“… After entering the school, my mentor advised me to study anatomy as soon as possible. An anatomy lesson is not something you would say I will study and comprehend the concepts overnight or for a week. You should attend the classes regularly. Students should provide accessible learning resources and not seek shortcuts…” " (Student* no. *1).*

Participants believed that successful students have good self-confidence and leadership in their learning process. Some students emphasized that the teachers can first build self-confidence and make them self-centered and self-regulated in learning. This concept requires the teachers to listen to them.

*“... If I realized the concepts satisfactorily, I would get a lot out of my effort. First, I accepted that anatomy is complicated, but I can help myself more than anyone. I started self-study. More action boosts self-confidence, which is required to become a doctor.“(Student* no. *6)*

Finally, the participants emphasized the importance of integration. Integration is simply defined as merging relevant content or subject areas. Teachers mention that the goal is to integrate and create a general perspective rather than a fragmented perspective of concepts. Some students believed that while multidisciplinary integration was provided, professors’ art of education, such as mastery of the teaching and learning process, generated the necessary harmony between the content presented. If well-coordinated, all aspects mutually reinforce each other, promoting learning in an integrated way.

*“... I enjoyed our integrated curriculum because that led to a better understanding of medicine. For example, discussing the anatomy of the gastrointestinal system gave me invaluable and integrated knowledge, and I enjoyed it ... “(Student* no. *4)*

Anatomy professors acknowledged that integration with clinical courses had changed their perspective, reflected in their teaching.

*“…During the integration process and while I was listening to the viewpoints of the clinical colleagues, I realized what I thought to be the crucial thing that students should all be aware of scarcely happens in the real world!...“ (FM* no. *2)*

2) **Mindful Clinical View**.

Mindfulness is defined as awareness of what is happening at the present moment. Some professors believed that they must be aware of the ultimate goal while teaching at each moment. Therefore, mindfulness is influenced by one’s dreams.

*“…I try to teach anatomy in a way that our current students and prospective physicians can diagnose heart enlargement in the future…” (FM* no. *7)*

If anatomy teachers gain a conservative view, the students can achieve integrity and professional identity as doctors.

*“... The student’s learning and professional identity depend largely on knowing and comprehending anatomy. Teachers can play the role of a good teacher in any situation, whether a crisis (like the Covid-19 pandemic) or not. A good teacher always applies the right approach....“ (FM* no. 1*).*

### Subtheme 2: role modeling and student support (heating up the students’ hearts)

The professors’ experiences in educating successful students during anatomy courses show that they should reduce unnecessary complaints and criticisms to penetrate their hearts and light the torch of knowledge in their minds. Feelings for the learners should be expressed, and love for them should be shown. There should be a role model for them in academia. If the love of science enlightens students, they are strengthened to endure hardships and problems. One of the professors said in this regard:

*“…As an ancient Persian anecdote, “to attract the heart of a friend, we must do every trick.“ I sat down during soccer World Cup games and watched part of the game with the students…” (FM* no. *8)*

### Joyful teaching (telling stories/games/humor)

According to the participants, turning hard work into enjoyable teaching and learning is the art of medical teachers. A few faculty members believe that humor and mixing lessons with fun and games can make students aware of their superior position as a professor. They find icebreaking essential at the beginning of each discussion.

*“…If students enjoy the class and assume that learning is a plausible activity, they will not waste their time. Most of the wasted time and students’ naughtiness occur because of the attractiveness of the classroom and teacher….“ (FM* no. *10)*

Such an approach strengthens the bond between the students and teachers and leads to effective role modeling.

*“... If the student does not follow me in the classroom, what is the benefit of my teaching class?“ (FM* no.*.8)*

Students can understand whether their teacher can be a trusted and knowledgeable role model and respond better if they encounter a fun and supportive teacher.

*“…good teachers are fully aware of all their behavior and movements and know what to teach in the classroom. They avoid traditional teaching methods and teach enjoyable for both students and teachers. Students will not be tired and enjoy the learning process….“ (Student* no. *10)*

### Student Support System

Both faculty members and students mentioned the student support system as influential. All the participants believe that student support is helpful for vulnerable students, those with learning disabilities, and successful and elite students. The faculty members referred them to counseling, helped them solve their educational and emotional problems, and supported their families emotionally and financially.

*“…Student support is essential, but it is not just for high-risk and susceptible students. Sometimes elites need even more assistance, and I have seen and experienced how sometimes talented kids are vulnerable...“ (FM* no.*8).*

Such support, mainly when anatomy professors are referred to as the first role models for medical students, motivates students to improve over the past.

*“…I had the support of the family and could work harder and enjoy learning; they did not hesitate to do anything for me. The teachers and faculty members were sometimes right supporters and solved my* problem*….“ (Student* no. *3)*

### Subtheme 3: sledgehammer approach (thorough engagement in the age of pandemics)

While anatomy is perceived as one of the most challenging courses for preclinical students, the advent of the Covid-19 pandemic adds to the challenge. Successful anatomy teachers state that they must apply a combination of educational theories quickly, seamlessly, and effectively in the classroom or online. They believe that teaching anatomy in pandemic situations should be accompanied by joy and vivacity and increase the moments of discovery and intuition for learning. The faculty members seem to struggle to get the most involvement from students.

Teaching approaches that trigger student involvement include a study guide, reference autonomy, neuroanatomical engagement, drawing, problem-based learning, and peer-based learning.

### Study guide

Most participants believe preparedness is an essential strategy for success in learning and teaching. The best way is to provide a friendly study guide. The study guide is like a travel guide designed by an instructor to guide learners. It can increase the student’s insight into the subject. Some professors had the experience of contributing to making “road map” books for anatomy. They believe that such a study guide is also a studying resource. Others found the study guide essential for managing the students’ learning.

*“... Student guidance is an important issue that needs to be planned and implemented. We need more insight and hard work in pandemic conditions. Our “road map” book that primarily serves as a reference of anatomy is a study guide as well…” (FM* no. *3)*

Students assume that having a study guide is an insightful experience with more potential than it seems. They argue that a good stud guide must include common mistakes and guide them in all learning experiences, especially in cadaver dissection sessions.

*“... The study guide helps a lot in learning, and it is excellent if students’ common mistakes are made in this lesson, for example, in the lab and when we dissect the cadaver ...“ (FM* no. *6)*

### Neuroanatomical engagement

Participants believed that people learned new abstract concepts better when presented in visual and auditory ways. Professors believed that due to the functions of the brain hemisphere, the information process occurred through two separate systems in the brain. The right half is dedicated to the image, and the left half is word processing. Visual learning complements auditory learning. Sensory learning, which happens by working on the cadaver, complements student learning. Such a multidimensional approach leads to long-term memorization.

*“…I always use fascinating slides and video clips. If necessary, I explain my experience through the film, which enhances the understanding and facilitates long-term memorization…” (FM* no. *5)*

### Reference autonomy

According to the participants, appropriate resources play an essential role in learning. A good resource should encourage the students to become more active and reinforce their thinking, reasoning, and comprehension. Resources must be dynamic to adapt in times of crisis (pandemic) and change to meet the needs of 21st-century learners using current technologies. There seems to be a demand for respecting autonomy in both student and teacher experiences. They react better when students feel free and supported to choose their resources.

*“…For anatomy, I use Gray’s Anatomy book. However, recently, one of my professors introduced Moore’s Anatomy book. I felt this book was better in the field of …” (Student* no. *2)*

Teachers argue that if provided with a dynamic curriculum design that encompasses clear goals, they can select from all the available resources and provide better ones. They believe that students should be taught to search and find valuable resources when they are highly interested in topics.

When teachers accept the principle of self-learning and self-guidance, they listen to their students feel more confident, leading to more enthusiasm and thus creating future lifelong learners.

*“…I had a student who came up to me and asked me to explain how to learn anatomy. I was surprised because I was unfamiliar with the question. I told him to get two books, look at them, and return them to me the day after. He came while he got many valuable points, and now he is looking for further resources. Now, he is one of my most successful students, and one of my most important duties is introducing excellent resources to students...“ (FM* no. *4)*

### Mindful dissection, better transition

The first official and scientific encounter of young students with the human body occurs in dissection sessions. The first sessions familiarize the students with the disgusting aspects of gross medical procedures in which teachers play a facilitating role in such transition.

*“…I did not feel good on the first day, and many of my friends could not see the cadavers. It smelled so bad, but when we got used to it and started learning, we had better feelings, especially when we had a practical examination …” (Student* no.*14)*

Anatomy teachers are the game changers who can seed respect towards the human body in students’ minds.

*“… A cadaver is the most important teaching tool in the anatomy course and serves as a teacher’s blackboard. It is full of teaching points for medical students. I think this is ethically important as well. They should learn to respect the dead body highly to show respect to the living body in the future….“ (FM* no.*12)*

### Drawing

Teachers and students have valuable experiences in learning and memorizing through drawing. Drawing helps them to understand the topic, consolidate what they have learned, and remember it in long-term memory.

*“…Anatomy has a regional nature. It means that, for instance, everything in the chest area should be learned simultaneously and as a whole. I use drawing to show the adjacency of structures so that students can better learn and consolidate the topic...“ (FM* no. *3)*

For some learners, drawing becomes a ritual; one of the talented students said:

*“…I always tell other students to draw pictures to understand anatomy correctly. Draw a lot! Anatomy cannot be understood without painting. No matter how strong you are in abstract thinking, still, draw! I had a notebook in which I was painting...“ (Student* no.*5)*

### Problem-based learning

Students will feel more satisfied if they are involved in future career problems. By using this approach, students are also emotionally involved in future challenges and are taught to enjoy solving future issues. Those problems that include detailed scene descriptions in a socio-emotional context and are related to cultural, moral, and social values seem to engage students better, help long-term memorization, and facilitate transition into future doctors.

One of the professors said:

*“… I will show the students a picture of the patient and tell them that the person has subarachnoid hemorrhage. Look at the patient’s family. You can see their deep worry. Tell me where the bleeding source is and what is the first step?... " (FM* no. *6)*

### Peer-assisted learning

Students stated that learning in small groups was attractive because they acquired interpersonal skills like listening, speaking, discussing, and group leadership. Besides, it promotes higher cognitive abilities such as reasoning and problem-solving.

*“...For me, the best learning tool is a peer to peer group work. I have good experience in all subjects from literature to anatomy utilizing this method…” (Student* no.*5)*

## Discussion

We first scrutinized the experience of teaching and learning anatomy through qualitative analysis. Next, we conceptualized a new approach to help medical students in anatomy courses have more satisfactory learning outcomes and a better transition into resilient future doctors. We called it “the Blacksmith Approach.“ building a sword (prosperous and resilient learner) takes three steps. 1. Creating a new forge (adequate preparation and mindful beginning) 2; heating students’ hearts (support and role modeling) 3. Sledgehammer’s approach (thorough engagement in the age of pandemics). (Fig. 1)

While other proposed educational methods in teaching anatomy focus mainly on learning outcomes during anatomy courses [14–19], the blacksmith approach tries to reach two goals, i.e., better teaching-learning outcomes and helping young students transition into future medical professionals. In fact, this is not another teaching method. It best serves as an educational framework for any pivotal, preclinical course capable of helping students acquire new roles and tackle challenges. In this sense, medical educators can adopt all prior teaching techniques as long as they help students reach better learning outcomes or have better transition experiences.

The blacksmith approach can be an educational implication of Schlossberg’s 4 S model for transition. Transition is a process that enables individuals to incorporate change into their life. The 4 S theory argues that an individual goes through a cyclic experience of 4 stages during the transition, i.e., situation, self, support, and strategies [46]. We encounter two types of transition in an educational setting. One happens within the learners and one within the educators. Although more attention is put on the former, we cannot achieve desired outcomes if we neglect the latter. While in the 4 S model, these four stages happen within the individual, the Blacksmith Approach creates a compatible, three-step learning environment for students and teachers to transition by creating a new attitude and mindset for both groups. As Browne et al. stated, individual motivation, background and circumstances, a sense of control, organizational support, effective networking, and information-seeking behavior contributed to a successful transition into and maintaining a strong self-identity as a medical educator [47].

### First Step: A new forge (mindset)

must be built for the students. This step is in line with the first stage of the four S model, i.e., Situation awareness. Here, the students become aware of what is happening. The teachers here have two roles. First, they should deliver the following messages to the students: (1) you are supposed to actualize a new identity (role change), (2) anatomy (as a pillar of medicine) provides a problematic task ahead (stress), and (3) you can help yourself more than anyone else. (control) Furthermore, the students should try self-study to increase their self-confidence (trigger). Secondly, the teachers should acquire a mindful clinical standpoint, i.e., developing good clinical knowledge and adopting active learning methods based on teamwork and clinical scenarios that encourage active participation and self-directed learning as if they are future doctors. This strategy has been shown to reduce student stress [11, 13] and help them react better as future physicians [3, 8, 48].

### Second step

The students’ hearts must be warmed up through support and role modeling. This step is compatible with the following two stages in the 4 S model, i.e., self and support. The students evaluate their demographic and psychological characteristics, and then they need to be aware of support systems and be able to get help [27, 29]. Here again, the role of anatomy teachers is critical. As medical students see anatomy as a gate to medicine, the anatomy teacher can be known as the gatekeeper. They will inevitably actualize their teachers as wise role models who are righteous supporters. The anatomy faculty member should be aware of their role modeling. Role modeling can help students foster resilience for future change, which leads to professionalism and integrity [7, 18, 49–52]. The teachers should adopt ice-breaking methods, use their sense of humor, tell stories, design games, and have friendly chats with students outside the classroom. In addition, anatomy teachers should gain the skills and knowledge for counseling and get familiar with support strategies to become prominent supporters of students [53–56].

### Third step: Sledgehammer’s approach

We found that, currently, preclinical students are facing two overwhelming stressors. On the one hand, the covid crisis has detached the students from their teachers and resources [15, 18, 57]. On the other hand, students need to handle their transition into knowledgeable medical professionals. Therefore we need to incorporate teaching methods that assist our detached students in coping with their stressors. The last stage in the 4 S model also deals with coping strategies. Students may use several coping strategies like information seeking, selective ignoring, and reframing the challenge [58].

Accordingly, the third step in our model includes three components; *thorough engagement, reframing the situation, and selective information seeking*. Regarding the first component, we encourage meticulously choosing more active and engaging learning techniques. Problem-based learning and Peer-based discussions in small groups, drawing, mind mapping, body painting, using play-doh, and interactive video content, are advised. According to Pandey and Zimitat (2007), a practical approach to anatomy education positively correlates with teaching and reflective learning quality. Successful anatomy learning requires balancing memorization with understanding and visualization[59]. Insightful experiences in teaching anatomy with this strategy are found in Australia (head and neck course) [15] and India (musculoskeletal anatomy) [13]. As Goodman et al. (2006) noted in their previous book, those with higher self-esteem and mental mastery are better able to cope with challenges. [60] A successful learning experience gives students a sense of control over their course, while applying a study guide, reference autonomy, and support system can help them gain self-esteem [61–63].

**Reframing** the situation, **As the second component**, is where the students need to redefine their roles and activities. Two sets of strategies can help students to reframe. 1) Using problem-based and peer-assisted learning with a flavor of sociocultural contexts can prepare students for better learning in a positive, non-intimidating learning environment to redefine themselves as future doctors for diagnosing and curing patients. Socio-emotional context and bringing drama elements into case scenarios have been recently emphasized to induce resilience and professionalism in medical students (2.14, 64).

Recent evidence indicates that gamification leads to positive learning outcomes, higher motivation, and engagement. However, several downsides should also be considered, including increased competition and task evaluation difficulties. Besides, it depends highly on the context in which the gamification is being implemented and its users [65–68]. Hamari et al. (2014) have reviewed empirical literature, showing that gamification has proven effective in many fields, most notably education. Studies in education and learning contexts illustrate how the gamification of learning outcomes has been positive by increasing motivation and engagement in learning and enjoyment tasks over time. However, the studies also highlighted some negative points that require attention, such as the effects of increased competition, task evaluation difficulties, and design features. The review indicates that gamification provides positive effects; however, the effects greatly depend on the context in which the gamification is being implemented and the users who use it [66]. Recent studies suggest that joy and humor facilitate a person’s transition into more socially severe roles (like becoming a doctor) [34]. Hence, teachers should use joyful techniques such as painting, ice braking, storytelling, gamification, dance, and music [4, 9, 15, 53].

The last component deals with **selective information seeking**. Students should have a study guide and be free to choose their desirable resources (anatomy reference books, digital resources, or web-based social media videos). We advise the medical school to design a dynamic curriculum encompassing clear goals. In this setting, the study guide can act as a tool for transferring core knowledge in anatomical sciences. As a result, the tedious aspect of bulky content in anatomy courses can be avoided, and the educational efficiency of synchronous and asynchronous learning can be increased in the pandemic era [55, 56, 61, 69, 70].

This research is in line with constructive learning theory that emphasizes student-centered learning, which states that students should be actively involved in education and contribute to knowledge creation [71]. In a special effort to create a modern educational curriculum at Stanford University, Prober et al. recommended building a framework of core knowledge, embedding it into rich interactive formats, and encouraging students to pursue knowledge in some deep, but not all, domains [63]. The Blacksmith approach facilitates personal and in-depth pursuits that help transfer students into lifelong learners and adapt to challenging situations [63, 64]. This statement is consistent with Wald and Monteverde (2021). They report that educational resilience is vital in supporting and sustaining healthcare professional identity development and facilitating the progress of students’ moral resilience and complexity, especially in times of pandemic [25].

According to the participants’ viewpoints, the applicability of the blacksmith approach is one of the most important aspects of improving the quality of anatomy teaching and learning in any situation, such as current critical conditions.

Our university’s online programs were adequately integrated into the curriculum design and used to guide students on a learning journey rather than merely as another resource; perhaps, these tools could be beneficial. However, in such circumstances, the epidemic and sanctions of Iran and the lack of resources, students and professors should take measures to optimize education to facilitate the teaching and learning of this course. Accordingly, from the above point, through a qualitative study, it was assumed that there were signs of successful teaching and learning and internal change in deep search among students and faculty members.

Revolution in medical education is a global program, and many medical schools have perceived investigation and experienced challenges [63, 64]. Correspondingly, an active and integrated strategy for teaching and learning anatomy has been emphasized in other medical education courses and areas, including a contemporary medical curriculum. Managers, practitioners, and professors can use the elements of this strategy to increase the efficiency of medical education, improve students’ knowledge, skills, and attitudes, and ultimately maintain and promote understanding of anatomy and other courses. Also, from the participant’s perspective, the applicability of students learning experiences and faculty members’ teaching experiences in the context and workplace conditions was one of the factors that changed the view of anatomy as an introductory course in medicine.

## Study limitation

As this study was conducted in one country, it is suggested that more studies and insights be obtained from other participants to increase the generalizability of findings. Besides, further work should be done to provide more practical guidance in the educational system regarding the transition of medical students in teaching and learning anatomy in the current pandemic crisis.

## Conclusion

Medical students experience a fundamental evolution into responsible and lifelong learners qualified as professional doctors. Educational systems focus primarily on teaching and learning, while students’ transition can be facilitated by a three-step model called the Blacksmith Approach, based on the study results. Initially, the blacksmith (educational system) must prepare a new forge. It means anatomy teachers should redesign the academic mentality of students by delivering messages about role change, difficulties ahead, and the importance of self-guidance, along with implementing clinical scenarios in active learning methods. Then, he/she must heat the metal. It implies that anatomy teachers, as gatekeepers of medicine, should adopt amusing teaching methods while embracing their role modeling and actively applying support strategies. Finally, he/she may shape the metal with a sledgehammer. Teachers need to use three components to create a sword (self-confident, accountable, resilient professional); thorough engagement by using active and engaging teaching techniques, reframing the situation with problem-based clinical scenarios, and joyful teaching. And selective information seeking via study guide and reference autonomy.

In conclusion, an active and integrated strategy for teaching and learning anatomy is suggested to be applied in other courses and areas of medical education in the current medical curriculum.

## Electronic supplementary material

Below is the link to the electronic supplementary material.

Supplementary Material 1

## Data Availability

The datasets produced and analyzed during the present study are not publicly accessible due to participant confidentiality but are obtainable from the corresponding author on reasonable request.

## List of abbreviations

**SUMS**: Shiraz University of Medical Sciences
**FM**: Faculty members
**MCQ**: Multiple-choice questions
**OSCE**: Objective structured clinical examination
