# Supplementary material for: The blacksmith approach: a strategy for teaching and learning in the medical anatomy course (a qualitative study)
Source: BMC Med Educ. 2022 Oct 20;22:728. doi: 10.1186/s12909-022-03800-1 (PMC9584281; doi:10.1186/s12909-022-03800-1)
Supplement: Supplementary file 1 — Supplementary Material 1 [file 12909_2022_3800_MOESM1_ESM.pdf]

Shiraz University of Medical Sciences

School of Medicine - Shiraz University of Medical Sciences

Research Ethics Certificate

Approval ID:	IR.SUMS.MED.REC.1397.490	Approval Date:	2019-01-29
Evaluated by:	School of Medicine - Shiraz University of Medical Sciences		
Status:	Approved		
Approval Statement:	The project was found to be in accordance to the ethical principles and the national norms and standards for conducting Medical Research in Iran.<br>Notice:<br>1. Although the proposal has been approved by the research ethics committee, meeting the professional and legal requirements is the sole responsibility of the PI and other project collaborators.<br>2. This certificate is reliant on the proposal/documents received by this committee on 2019-01-29. The committee must be notified by the PI as soon as the proposal/documents are modified.		
Thesis Title:	Evaluation of Successful strategies in teaching and learning activities in anatomy based on the experiences of teachers and students		
Thesis Adviser (PI):	Name: Leila bazrafcan<br>Email: bazrafcan@gmail.com		
Student:	Name: Arash Shojaee<br>Email: Arashshojaei4030@gmail.com		

Dr. Mohammad Javad Ashraf
Director of Institutional Research Ethics Committee
School of Medicine - Shiraz University of Medical Sciences

Dr. Mohammad ali Sahmeddini
Secretary of Institutional Research Ethics Committee
School of Medicine - Shiraz University of Medical Sciences
